# A Plaque-Like CD34-Positive Dermal Fibroma Presenting as an Acquired Papule

**DOI:** 10.7759/cureus.39231

**Published:** 2023-05-19

**Authors:** Alpa Kanji, James Carton, Caroline Hewitt, Derrick Phillips

**Affiliations:** 1 Dermatology, Imperial College Healthcare NHS (National Health Service) Trust, London, GBR; 2 Histopathology, Imperial College Healthcare NHS (National Health Service) Trust, London, GBR

**Keywords:** cytogenetic studies, benign lesions, spindle cell, fibroma, dermatofibrosarcoma protruberans

## Abstract

CD34-positive dermal fibromas (PDFs) are cutaneous neoplasms that display a characteristic pattern of superficial dermal spindle cell proliferation on histopathology evaluation. They are clinically heterogenous in presentation and thought to follow a benign course. CD34-PDFs have features that overlap with dermatofibrosarcoma protuberans (DFSP), a locally aggressive low-grade superficial sarcoma. Cytogenetic studies are essential to distinguish the two. This report presents the case of a 38-year-old female with a CD34-PDF on the right antecubital fossa.

## Introduction

A CD34-positive dermal fibroma (PDF) is a rare and poorly characterised benign lesion which can be congenital or acquired. This was first described in 2004 by Rodriguez-Juardo et al. [1], and since then there have been further reports of entities which are similar in terms of their histopathology findings but their clinical features can be very variable. CD34-PDF can mimic other lesions; thus there is a wide differential diagnosis. As it typically follows a benign course but can be mistaken for other entities on a superficial biopsy, unnecessary interventions may be deployed. One possible differential diagnosis is dermatofibrosarcoma protuberans (DFSP) and therefore correct and early identification is important.

We report the case of a 38-year-old female who presented with an acquired papule on the arm. Histological evaluation of a skin biopsy established this was a CD34-PDF. We have differentiated this from DFSP using cytogenetic studies.

This case report was previously presented as a poster at the 31st European Academy of Dermatology and Venereology (EADV) Congress Meeting on September 7-10, 2022.

## Case presentation

**Figure 1:**
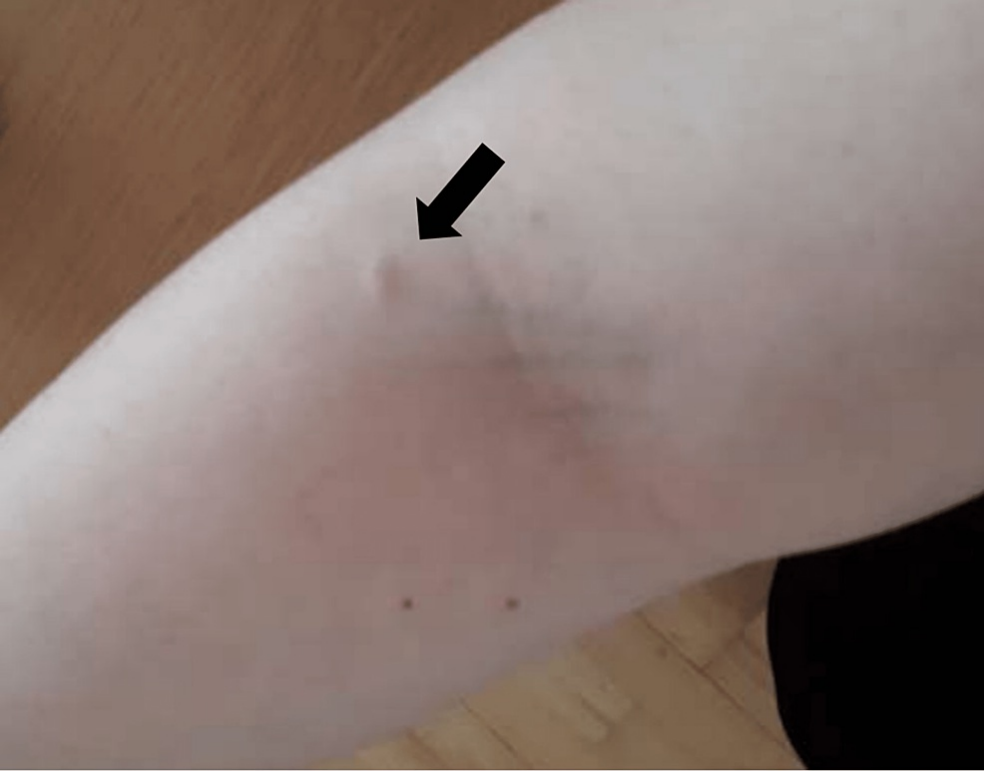
An image of the pea-sized dermal papule on the right antecubital fossa

We report the case of a 38-year-old female presenting with a 12-month history of a pea-sized papule on the right lateral antecubital fossa. She had a background of sarcoidosis, which was in remission. The lesion was asymptomatic and failed to respond to topical corticosteroids. On examination, there was a firm, mobile, non-tender dermal papule (Figure 1).

**Figure 2:**
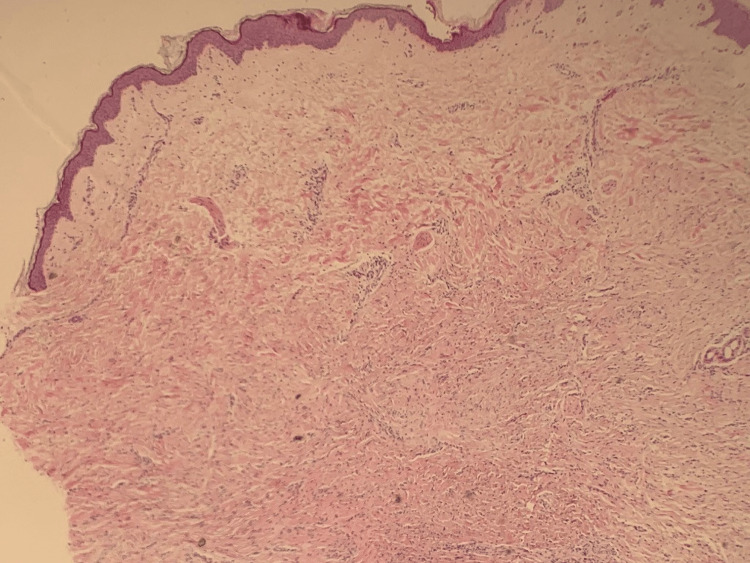
Lesional histopathology; Haematoxylin and Eosin x 40 magnification

**Figure 3:**
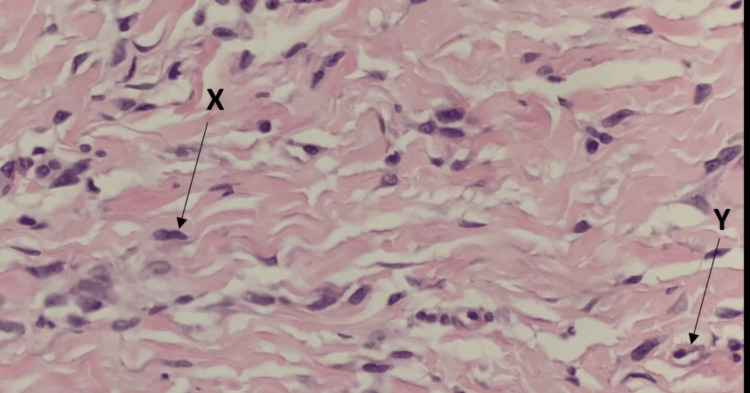
Histopathology showing a spindle cell proliferation in the deep dermis admixed with blood vessels; Haematoxylin and Eosin x 400 magnification Arrow X shows a spindle cell; Y shows a blood vessel

**Figure 4:**
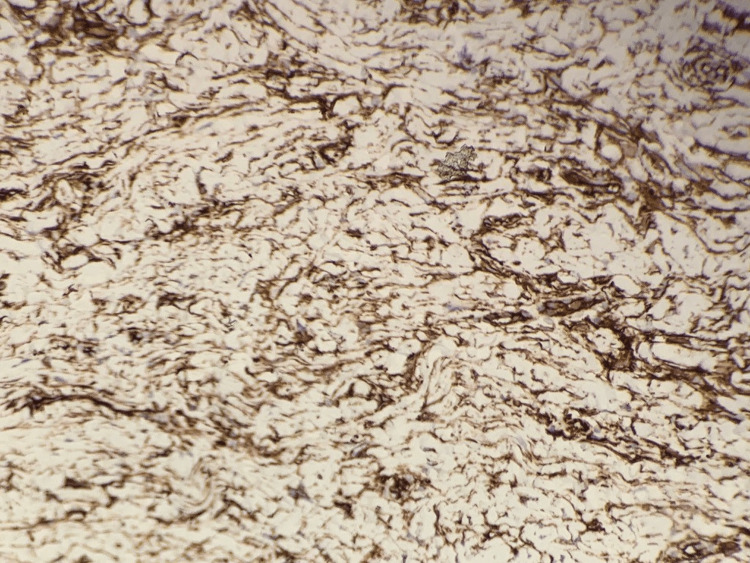
CD34 immunostaining x 100 magnification

The lesion was removed with a punch biopsy, and histological analysis showed a benign-appearing spindle cell proliferation in the deep dermis with admixed compressed thin-walled blood vessels (Figures 2-3). Immunohistochemistry was performed to further characterise the tumour: spindle cells stained positive for CD34 (Figure 4) with some focal expression of factor XIIIa, smooth muscle actin (SMA), and CD68. There was no reactivity for S100, MNF116, CD31, desmin, and epithelial membrane antigen (EMA). Mib1 proliferation was very low.

Overall, the findings were in keeping with a plaque-like CD34-positive dermal fibroma, a cutaneous neoplasm composed of CD34-positive dendritic cells. This entity has also been referred to as medallion-like dermal dendrocyte hamartoma (MDDH) [1].

## Discussion

PDF was first reported in 2004, occurring as congenital atrophic, well-circumscribed patches on the neck and upper chest of three young females [1]. Subsequent reports demonstrate that plaque-like CD34-PDF represents a clinically heterogeneous group with significant variability in clinical features though characterised by common histopathological findings. It has since been reported to be symptomatic, acquired (thus can present at any age) and present on the extremities [2]. As in our case, there have been other reports of CD34-PDF presenting as a dermal nodule with no epidermal change [2]. Initially, factor XIIIa was thought to be a marker for CD34-PDF but this has not been consistent in subsequent reports [3-5].

The common histological finding of this entity is a pattern of superficial dermal spindle cell proliferation with mostly horizontally oriented tumour cells [3]. Differential diagnoses of CD34-PDF include a variety of superficial dermal fibroblastic proliferations including dermatofibromas [3] or a diffuse neurofibroma [6], thus obtaining a skin biopsy is important. Perhaps the most important consideration is that of DFSP, which may have overlapping clinical features [4]. As the latter is a locally aggressive tumour, it is critical to distinguish between the two, particularly as the treatment of DFSP requires surgical management. In contrast, CD34-PDF is thought to follow a benign course, thus management tends to be conservative. Therefore, early recognition is important to avoid unnecessary surgical intervention. 

Where it is difficult to distinguish the two, cytogenetic studies are essential to test for the t(17;22) (q22;q13) fusion gene of DFSP using probes for COL1A1 and platelet-derived growth factor beta (PDGFB), which is present in DFSP [4]. In our case, cytogenetic analysis to identify the COL1A1-PDGFB fusion was negative, thus excluding the possibility of DFSP.

## Conclusions

We report the case of a plaque-like CD34-PDF, which is an interesting yet rare entity. Further studies are needed to learn more about this neoplasm and to fully define its diagnostic spectrum, classification, and pathology. Our case adds to the limited reports of this entity published to date. We continue to monitor our patient in lieu of excision.
